# The Effect of Climate Fluctuation on Chimpanzee Birth Sex Ratio

**DOI:** 10.1371/journal.pone.0035610

**Published:** 2012-04-26

**Authors:** Hjalmar S. Kühl, Antoine N'Guessan, Julia Riedel, Sonja Metzger, Tobias Deschner

**Affiliations:** 1 Department of Primatology, Max Planck Institute for Evolutionary Anthropology, Leipzig, Germany; 2 Université de Cocody, Abidjan, Côte d'Ivoire; 3 Centre Suisse de Recherches Scientifiques en Côte d'Ivoire, Abidjan, Côte-d'Ivoire; 4 Robert Koch Institute, Berlin, Germany; University of Turku, Finland

## Abstract

Climate and weather conditions, such as the North Atlantic Oscillation, precipitation and temperature influence the birth sex ratio (BSR) of various higher latitude species, including deer, elephant seals or northern human populations. Although, tropical regions show only little variation in temperature, climate and weather conditions can fluctuate with consequences for phenology and food resource availability. Here, we evaluate, whether the BSR of chimpanzees, inhabiting African tropical forests, is affected by climate fluctuations as well. Additionally, we evaluate, if variation in consumption of a key food resource with high nutritional value, *Coula edulis* nuts, is linked to both climate fluctuations and variation in BSR. We use long-term data from two study groups located in Taï National Park, Côte d'Ivoire to assess the influence of local weather conditions and the global climate driver El Niño Southern Oscillation (ENSO) on offspring sex. Côte d'Ivoire has experienced considerable climate variation over the last decades, with increasing temperature and declining precipitation. For both groups we find very similar time windows around the month of conception, in which offspring sex is well predicted by ENSO, with more males following low ENSO values, corresponding to periods of high rainfall. Furthermore, we find that the time spent cracking and feeding on *Coula* nuts is strongly influenced by climate conditions. Although, some of our analysis suggest that a higher proportion of males is born after periods with higher nut consumption frequency, we cannot conclude decisively at this point that nut consumption may influence shifts in BSR. All results combined suggest that also chimpanzees may experience climate related shifts in offspring sex ratios as response to climate fluctuation.

## Introduction

The effects of short-term climate variability, which include changes in precipitation patterns and resource availability, unusual temperatures, and extreme weather conditions, affect the behavioral, physiological, and demographic responses of a multitude of species [1], [2]. Human-induced long-term climate change enhances these effects dramatically [2], [3]. How a species copes with the consequences of climate variation is generally related to its generation time, distributional range, and population size, as well as its physiological and behavioral flexibility [4]. Species may further respond with phenotypic plasticity [5], behavioral variation or genetic adaptation [6], but may also be limited in their capacity to adapt to changed conditions. Therefore, ongoing and accelerating climate change is considered to be an additional threat to and a major concern for the conservation of many endangered species with small, fragmented populations or limited ranges [7]. Predictions of when and how a species will be affected by altered conditions can be formulated with regard to all levels of organismic life, such as offspring survival [8], range shifts [3], migration [9], physiological adaptation to altered resources [10], social interactions [11], disease dynamics [12], and birth sex ratios [13].

The study of birth sex ratio (BSR) biases is appealing in this context as it can be grounded in a well-developed theoretical concept from evolutionary biology of why and when species should adaptively bias offspring sex ratio in response to social, demographic and environmental conditions. This includes for example the facultative adjustment of BSR in relation to maternal conditions, “local resource enhancement” with BSR bias towards the more helpful sex or “local resource competition”, when females bias offspring BSR towards the dispersing sex to reduce existing competition over local resources [14]–[17]. It also provides the opportunity to study biased BSR as constraints in the context of harsh environmental conditions [18].

Several recent studies have established clear links between offspring BSR and the influence of local weather conditions, such as precipitation and global climate drivers. Male-biased BSR has been observed during warmer years in historic human populations [19]. In red deer (*Cervus elavus*) [20] fewer males are born with increasing winter rainfall. In elephant seals (*Mirounga angustirostris*) [13], BSRs have been closely linked to the North Atlantic Oscillation (NAO), a climate driver on the northern hemisphere, with more males being born at low NAO values. Most of the studies establishing links between BSR and climate variation have focused on higher latitude species. Although tropical and subtropical species have been the focus of many studies on BSR biases, previous work has not considered climate fluctuation as a potential driver of BSR biases.

Here, we present a study on chimpanzee BSR variation in relation to climate fluctuation using long-term data on two study groups in Taï National Park (TNP), Côte d'Ivoire. Unlike many other primate species, chimpanzees are not known to exhibit considerable species-level variation in BSR [17]. However, Côte d'Ivoire has experienced extensive climate fluctuations over the last few decades, with a general decrease in precipitation and increase in temperatures since the 1960s [21]. Furthermore, parts of West Africa and the Taï region are known to be affected by El Niño Southern Oscillation (ENSO), a global climate driver affecting for example local rainfall patterns and temperature [22].

We start by assessing variation in local rainfall at the study site and its association with ENSO. Second, we evaluate the relationship between ENSO and offspring sex, predicting that relatively more males should be born after periods, which are preceded by low ENSO conditions corresponding to high rainfall.

Also, if climate conditions influence BSR via nutritional status of females and food availability, as has often been proposed, one should expect to find considerable variation in chimpanzee food resources and BSR should in turn be linked to this variation [23]. However, studying food availability and food intake as a function of climate variation is a project in itself. Here, we just focus on one example, the consumption of the nut *Coula edulis*. These nuts are a preferred food item and of particular nutritional value, containing a high proportion of unsaturated fatty acids [24]. Nut cracking is a characteristic behavior in the Taï chimpanzee population, not found in most other chimpanzee populations. We relate variation in the time spent nut cracking and eating to both ENSO variation and BSR and expect a higher proportion of male birth, when more nuts are available.

Variation in Taï chimpanzee offspring sex is closely related to fluctuations in ENSO and local weather conditions, with the probability of male birth being higher at low ENSO and high rainfall, respectively.

## Methods

### Field data collection

#### Study groups

The two chimpanzee study groups are situated in TNP, Côte d'Ivoire, and are referred to as North and South group, respectively [25]–[26]. Until recently, they were separated by another small chimpanzee community, which has now disappeared. The closest distance between their homerange borders is about 2 km. Although, consisting of largely pristine forest the territory of the North group was affected by logging and forest encroachment in the 1970s. The territory of the South group was also affected, but to a lesser extent and consists largely of primary rainforest [27].

The BSR data for this study come from the long-term data collection of the Taï Chimpanzee Project; for the North group, 92 births were recorded between 1982 and 2008, and for the South group, 50 births were recorded between 1995 and 2008. In the early phase of the project (1982–1986), nine newborn infants in the North group died before their sexes could be identified. This reduced the available number of sexed newborns in the North group to 83. In addition, the birth month of another nine individuals, could not be reliably assigned. Throughout this manuscript, BSR refers to the proportion of male births, defined as the number of males born divided by the number of all infants born.

Consumption of *Coula* nuts was approximated by the time spent cracking and eating nuts. This data was collected during daily focal follows of individuals. For North group this information is available from 1992 and for South group from 1999 onwards. However, in the 2000s data collection was interrupted repeatedly because of ongoing civil war in the country. This limited us in using time series of both groups and conducting analysis for both groups in the same way.

### Analytical methods

#### Climate variables

Rainfall and temperature data were collected since 1987 at the study site, however, these time series were incomplete due to repeated interruption of work in periods of civil war in the country. Additionally, available interpolated rainfall data did not correlate well with recorded local rainfall. Therefore, we could not base our analyses solely on these data. Instead, we used an index of El Niño Southern Oscillation (ENSO), the Multivariate El Niño Southern Oscillation Index (MEI) to conduct most of our analyses (http://www.esrl.noaa.gov/psd/people/klaus.wolter/MEI/). ENSO is a global climate driver for which time series data is available since many decades. The use of ENSO/MEI is well justified as it influences local whether conditions, such as temperature and rainfall in West Africa [22]. Furthermore, a clear link between ecological phenomena and climate indices has been demonstrated in many studies [28]. The available local rainfall data we used for verifying its relationship with MEI, as well as for establishing its relationship with BSR for a limited period. Similarly, we used the normalized Difference Vegetation Index (NDVI, http://www.landcover.org), a measure of canopy greenness [29] as an additional variable to demonstrate its relationship with BSR for a limited period.

#### BSR and time lags

**Figure 1:**
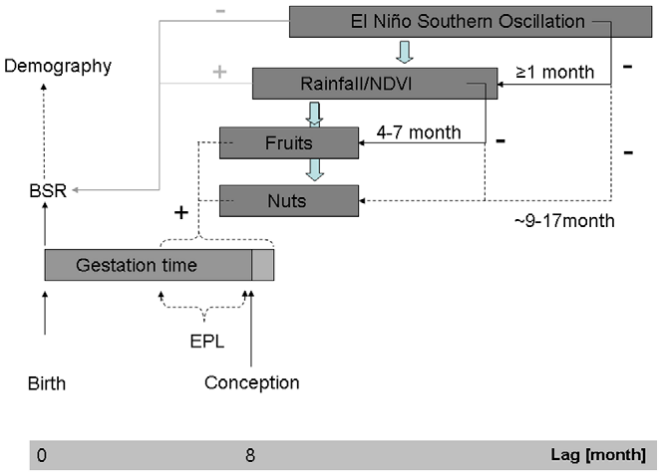
Schematic representation of climate-food and BSR relationships. Correlations found previously are represented as solid black lines and those hypothesized for this study as dashed black lines. The correlational relationship between ENSO, rainfall, NDVI and BSR is indicated by grey lines. Plus/minus symbols indicate directions of relationship, for correlations with BSR they refer to male bias. EPL-Early pregnancy loss could be one potential BSR determining mechanism, however, other mechanism are possible as well (see discussion).

ENSO influences local rainfall and whether conditions with a delay of one to several months [22]. Fruiting phenology at Taï lags behind rainfall patterns with a suggested four - seven month period [30]. NDVI reflects water stress and is therefore a direct proxy for vegetation conditions with no lag [29] (see also Fig. 1).

Consequently, one can hypothesize at least two critical time periods, in which an influence of climate conditions on offspring sex is likely to occur. The first critical time window covers the months around conception. Weather conditions might directly influence offspring sex determination at conception, e.g. through variation in mating behavior or physiological status of females [31] or soon thereafter through differential fetal loss [20], [32]. A second critical time window can be expected to exist several months before conception, during flowering or fruit maturation. Weather conditions determining fruit production and maturation, as found in previous studies [30], may eventually influence mating behavior and the physiological status of females around the time of conception or soon thereafter (see also Fig. 1).

#### Model fitting

The discrete probability *P* of observing *n* male births given a total number of *N* births over the study period is
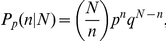
with *p = 0.5* for a male birth in the non climate effect model and 
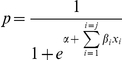
 in the climate effect model. *q = 1−p* is the probability for a female birth, α is the intercept and *β*_i_ are the coefficients for the climate variables *x_i_*. Assuming an influence of MEI at a given time lag (*l_t_*) and influential window (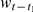) on birth sex probability without any seasonal effect, *p* becomes 
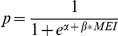
.

Given the hypotheses formulated prior to analyses, we evaluated models for the birth data of both communities with MEI time lags for *l_t_* = 6–18 months and influential windows of w_t-t1_ = 1–6 months.

Initially we also included an autocorrelation term into the model. Autocorrelation would arise, if offspring born close by in time has the tendency to have more often the same sex compared to offspring born during other periods and if this tendency is not explained by other variables in the model. This would violate the assumption of independent residuals.

For this, we first evaluated a Generalized Mixed Model (GLMM; [33]) including ‘MEI’ as fixed effect and ‘offspring mother’ as random effect to derive residuals. We then calculated for each data point the autocorrelation term by averaging the residuals of all other data points weighted by the time between births. The weight followed a normal distribution. We estimated the standard deviation by minimizing the AIC of the GLMM including the calculated autocorrelation term as additional variable. However, the inclusion of the autocorrelation term did not show the presence of positively correlated residuals. Additionally, we also evaluated, if the sequence of male and female births, respectively, would be more clumped than expected by chance. For this we conducted a runs test, which evaluates the randomness in a two valued data sequence [34]. There was no evidence to assume that the birth data would show a particularly clumped distribution (p = 0.08), rather it indicated a trend towards over-mixing of male and female births, which is the opposite of what one would expect to find, if positive autocorrelation played a role. We therefore excluded it again from the final GLMM that only included ‘MEI’ as fixed effect and ‘offspring mother’ as random effect.

Last, we ran a model to also evaluate the relationship between the proportion of time spent nut cracking and eating and offspring sex. Here, we concentrated on the time around conception, as any positive influence of nut consumption on offspring sex through female physiological status should have the greatest effect during this period. We used maximum likelihood methods to estimate model. For the issue of multiple testing, please see discussion. All analyses were done in R using the functions ‘lmer’ and runs.test [35].

## Results

### Local rainfall and MEI variation

**Figure 2:**
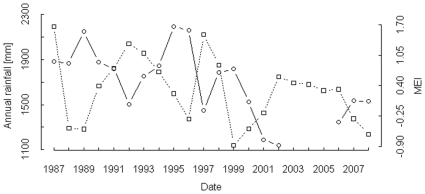
Annual rainfall at Taï study site and MEI “Multivariate El Niño Southern Oscillation Index”. For the period from 2003–2005, rainfall data (open circles) were only partially available and were thus omitted.

Rainfall at the Taï study site was strongly negatively correlated with MEI (Fig. 2). The annual rainfall measured at Taï since 1987 decreased significantly throughout this period, with an average yearly decline of about 29.6 mm. This resulted in about 500 mm (∼27%) less rainfall for the recent years compared to the early phase (y = −29.577x+60753, R^2^ = 0.38, p<0.01). In addition, rainfall varied considerably throughout this period with a more than 50% difference in the amount of annual rainfall.

### Offspring sex and climate variation

In North group, slightly more females (45) than males (38) were born during the study period, while in the South group, a few more males were born (27) than females (24). However, overall BSR bias did not significantly deviate from the expected 1∶1 male-female ratio in either the North (binomial test, N = 83, t = 0.46, p = 0.51) or South group (binomial test, N = 51, t = 0.53, p = 0.78).

**Figure 3:**
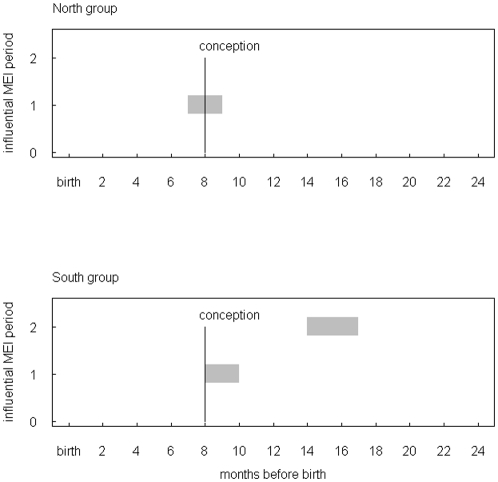
Influential MEI time windows on offspring sex. North (above) and South group (below). For both groups a potentially influential MEI time window was identified for the time around conception, for South group a second period emerged about 13 months before births.

**Figure 4:**
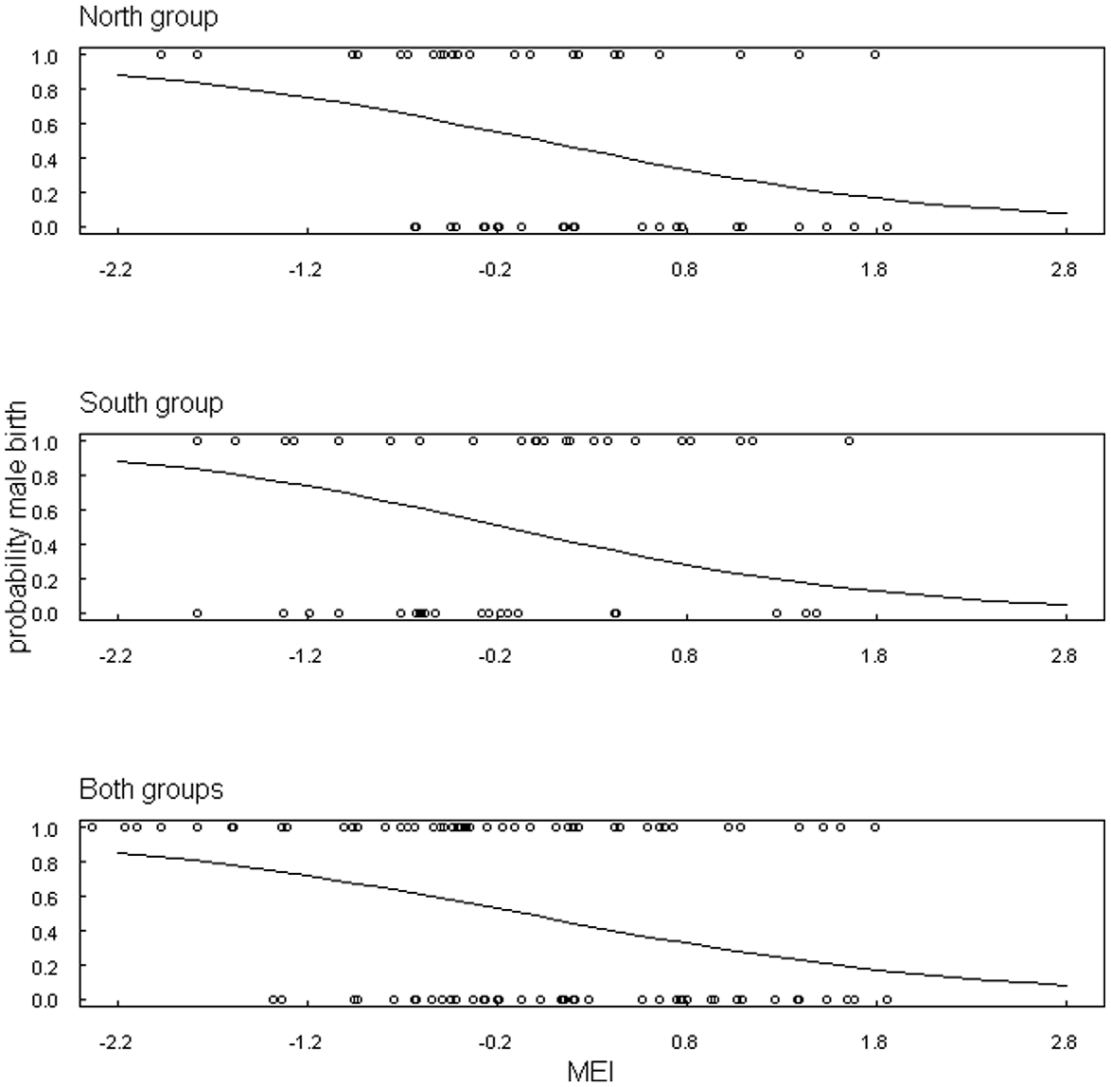
Model predictions for probability of male/female birth based on MEI. The prediction refers to the identified time period around conception (lag 7 months, time window 2 months). Male and female births are represented as circles at 0 and 1, respectively. Please note that circles representing births with same preceding MEI values overlap completely.

**Figure 5:**
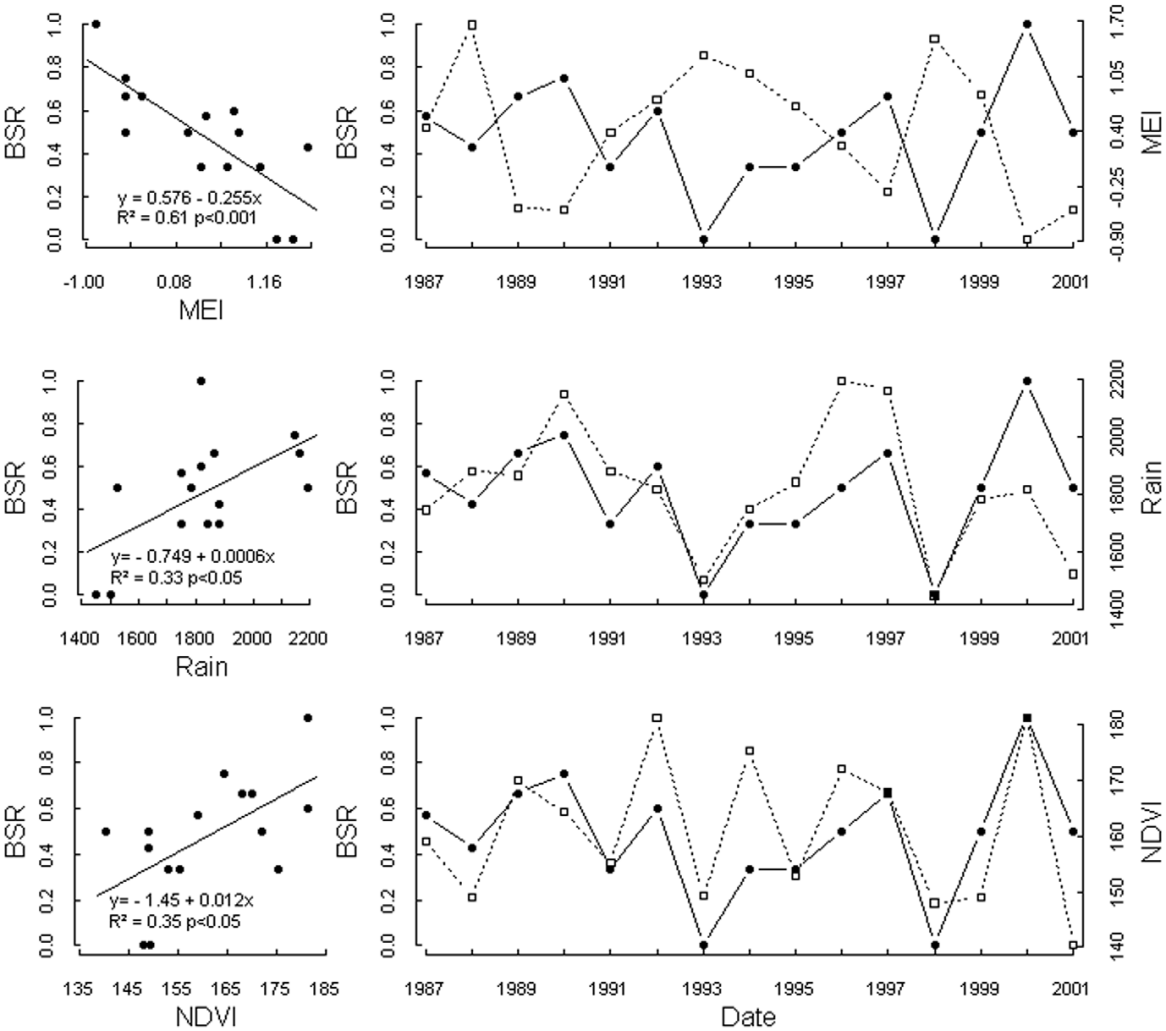
Annual BSRs of North group plotted against rainfall (above), MEI (middle), and NDVI (below). The solid line with filled circles represent the BSR data, the dashed line with open squares represents the respective climate/weather time series. On right side of the panel, time series of annual BSRs of North group (1987–2001) and rainfall, MEI, NDVI, respectively.

The correlation between MEI and offspring sex revealed for both chimpanzee groups almost identical influential time windows (Fig. 3, 4). In both groups a significant relationship between MEI and offspring sex was found for a time window around the month of conception (Table 1). For south group a second window was found lagging conception and birth by 5–8 and 13–16 months, respectively. This variation in offspring sex as a function of climate variability was supported by the relationship between BSR and local rainfall and NDVI for the limited time period for which these data were available (Fig. 5).

**Table 1:** Results from GLMM analysis for North and South group.

group	model	lag/window	par	AIC	int	mei
North	mei	6/3	3	70.6	0.004	0.83*
North	mei	7/2	3	68.9	−0.03	0.91*
North	null		2	73.2	0.08	
South	mei	7/2	3	69.2	0.15	1**
South	mei	13/4	3	70.6	−0.17	−0.58*
South	mei	13/5	3	70.8	−0.17	−0.55*
South	null		2	73.2	−0.08	
Both	mei	7/2	3	133.3	0.05	0.84***
Both	null		2	142.6	0.002	

Models evaluated included an index of El Niño Southern Oscillation ‘mei’; for each model evaluated the ‘lag/window’ are given in months; the number of parameters ‘par’ including both fixed (i.e. MEI) and random effects (i.e. offspring mother): AIC; the parameter estimates for the intercept ‘int’, ‘mei’(* indicates significance on 0.05 level, ** on 0.01 level, *** on 0.001 level). A negative ‘mei’ parameter estimate indicates a higher probability of male birth at high MEI values. Please note that the AIC values are derived from restricted maximum likelihood estimation and represent therefore only approximations. The ‘null’ model has no additional parameter. As the time lag of seven months with an average MEI value (window) of two months is shared by both groups, the GLMM results for the analysis combining both groups are shown as well.

### Climate and spatio-temporal variation in nut cracking

**Figure 6:**
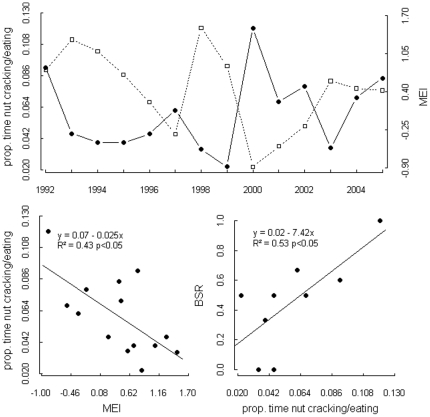
MEI and proportion of time spent nut cracking in North group. As time series (above), and scatterplot (below left), (below, right) proportion of time spent nut cracking and eating in north group at a time lag of 12 months and annual BSR.

The temporal variation in fruiting patterns of key food species is driven by climate and local weather conditions and generates clear signals in the consumption frequency of available resources. Feeding on *Coula edulis*, measured as the time spent cracking and eating these nuts, was highly correlated with MEI (Fig. 6a,b) and lags 9–17 months behind MEI (linear regression, for all lags 9–17, p<0.005). The analysis of nut consumption and offspring sex provided varying results. Whereas the annual BSR and proportion of time spent nut cracking and eating indicated that more males were born in periods preceded by high nut consumption at a one year time lag (Fig. 6c), the results from logistic regression analysis were less clear. Identified time lags between nut consumption and birth also suggested a higher probability of male birth for the months 11–13 similar to the annual analysis. However, an additional potentially influential time lag appeared at month seven and eight with a higher probability of female birth.

## Discussion

The finding of our study is that chimpanzee offspring sex can be influenced by climate and weather conditions, at least in the study population. This is remarkable, since tropical forests are assumed to be a quite stable environment, compared to those of higher latitude species, for which climate and weather dependent shifts in BSR have been reported previously. Although we evaluated the availability of only one key food item, *Coula* nuts, as a function of ENSO variation, the found ENSO - nut cracking and eating relationship might indicate that chimpanzees live in a much more variable environment and are more affected by it than previously thought.

Unfortunately, we had only a relatively small dataset with 142 births available and conducted a multiple testing exercise to establish the ENSO-offspring sex relationship. This is the critical and weak point of our study. However, we are positive that our results are not just an artifact of these limitations for several reasons. First, both study groups show very similar ENSO-offspring sex relationships with regard to the first time window identified for the months around conception. Furthermore, the time series data used for both groups overlapped only partially (North group: 1987–2001; South group: 1995–2008). Therefore, the observed pattern is highly unlikely to occur, if observed relationships were purely at random. Second, only two influential ENSO-BSR time windows were identified per group, respectively, corresponding well to expected sensitive periods: the time around conception (both groups) and 5–9 month before (only South group), when climate and weather conditions are known to influence food availability several months later [30]. Third, the ENSO-offspring sex relationship is supported by additional analyses, such as the observed rainfall variation at the study site, the rainfall-ENSO relationship, and consequently also the relationships between BSR, rainfall and NDVI, respectively.

Assuming that our findings are indeed correct, one needs to ask questions about potential mechanisms. Although, it is quite possible that different mechanisms may play a role, early pregnancy loss (EPL) associated with differential fetal survival may be one likely explanation. Various studies in humans and animals have shown that stress induced by environmental conditions during the early phase of pregnancy, such as weather conditions, nutritional status or physical strain can lead to EPL [20], [23], [36]. Additionally, because local weather conditions are tele-connected to ENSO with a time lag of at least one month, the influential ENSO window around the time of conception is likely to influence on site rainfall also some time after conception. Obviously, more in depth studies on female physiological status, hormone levels and abortions are needed to draw definitive conclusions about BSR determining mechanisms.

In this context the difference in fruit availability between the two groups is also worth to be discussed [37]. South group has a fruit availability index (FAI) about twice as high as North group. This difference could be a possible explanation for the emergence of a second MEI period about one year ahead of births which is positively correlated with a higher proportion of male births. This time window we found only for South group. As previously found the FAI is negatively influenced by the amount of rainfall in the preceding 4–7 months [30]. Thus the higher MEI, the lower the amount of precipitation and consequently the higher the FAI around the time of conception. According to BSR theory these more suitable conditions should then favor a higher proportion of male births. However, at this point it is mere speculation whether difference in food availability and MEI-offspring sex between the groups are related. Further in depth studies are needed that try to replicate and to better understand our findings.

The results on the one food item, we looked at more in detail, nuts, need also some discussion. Nut availability varies greatly between years, furthermore, nuts have high nutritional value, in particular they have a high proportion of unsaturated fatty acids, which play a major role in many physiological and biochemical processes [24]. Variation in nut availability seems to be highly dependent on climate fluctuations, which in our study we approximated by ENSO. However, the multiple correlation results we got when analyzing the relationship between nut consumption and offspring sex is puzzling and may just indicate effects of the small dataset we had available and the inherent multiple testing problem. Any further interpretation and conclusion at this point is mere speculation and a larger dataset with physiological information is needed to evaluate this hypothesis more rigorously. Nevertheless, one should emphasize that this issue does not affect the established climate-offspring relationship, it only limits us in drawing more detailed conclusions about a potential mechanism of BSR determination.

Eventually, it is also interesting to consider potential consequences of observed climate-offspring sex relationships. Effect sizes of shifts in BSR are often rather small, with no longer term consequences for the population dynamics of a species. In other cases observed shifts may indeed lead to a pronounced bias in the operational adult sex ratio with consequences for various behavior or population dynamics [1]. At this point it is also difficult to say, whether the observed variation in chimpanzee offspring sex are a consequence of normal climate and weather fluctuations, which have existed already before human-induced climate change or whether the apparent change in Côte d'Ivoire's climate over the last decades has been the major driver of the observed phenomena.

In conclusion, our work provides a new perspective on the study of variation in offspring sex in chimpanzees and possibly variation in other tropical species. The influence of climate and weather conditions seems according to our results not limited to higher latitude species and other tropical species may show similar patterns. Care should be taken not to over interpret our results, but to see the value of this study in generating several hypotheses that could probably be addressed using available extensive field data from the various long-term ape and primate field sites, with the collection of some additional data related to nutritional value of resources and food intake, physiological status of females and mating behavior.
